# A Bilateral Wolffian Adnexal Tumor with Malignant Behavior: A Rare Case Report with Literature Review

**DOI:** 10.30699/IJP.2024.1999797.3095

**Published:** 2024-10-29

**Authors:** Shabnam Mashhadi, Fereshteh Ameli, Shahrzad Sheikhhasani, Sara Parviz, Fatemeh Nili, Behnaz Jahanbin, Arezoo Esmailzadeh

**Affiliations:** 1 *Department of Pathology, Cancer Institute, Imam Khomeini Hospital Complex, Tehran University of Medic al Sciences, Tehran, Iran *; 2 *Gynecology Oncology Department, Vali-e-Asr Hospital, Tehran University of Medical Sciences, Tehran, Iran*; 3 *Department of Radiology, School of Medicine, Imam Khomeini Hospital, Tehran University of Medical Sciences, Tehran, Iran*; 4 *Department of Obstetrics and Gynecology, Trauma Research Center, Baqiyatallah University of Medical Sciences, Tehran, Iran*

**Keywords:** Bilateral Wolffian, Adnexal Tumor, Malignant Behavior, Case Report

## Abstract

Wolffian adnexal tumors (FATWOs) originate from the mesonephric duct remnants. FATWOs are extremely rare and 100 incidental FATWOs have been reported in the English literature as of now. Most FATWOs have low potential for malignancy but aggressive behavior including recurrence and metastasis have been described in few cases; There is no standard protocol for optimal treatment of FATWOs. The case described here is a 35-year-old female who presented with a right-side ovarian mass via abdominal ultrasound. She had a history of left salpingo-oophorectomy due to an abdominal mass, which both histopathologic and immunohistochemical study’s findings were consistent with Wolffian tumor. Later, she underwent total abdominal hysterectomy with tumor debulking because of the probable malignant behavior of the tumor. FATWO has a heterogeneous histologic pattern which may make its diagnosis challenging. No specific immunohistochemical markers have yet been recognized for FATWO and pathogenesis or molecular alterations are not definitive. Therefore, there is no comprehensive recommendation for optimal clinical management of FATWO or its recurrence.

## Introduction

The term of “Female adnexal tumor of probable Wolffian origin” (FATWO) initially was described by Kariminejad and Scully in 1973, and its immunohistochemical, as well as electron microscopic findings, were reported afterward (1). They described FATWO as a benign tumor despite its mitotic activity and capsular invasion (2). Although most FATWOs are asymptomatic and the tumor is usually incidentally identified, patients with FATWO may suffer from abdominal pain or mass (3). Origin of FATWO is the broad ligament, which is the remnant of the mesonephric duct that is completely distinct from Mullerian structures. The mesonephric system is the origin of various organs, including the broad ligament, which is a common location for FATWO. Mesosalpynx, fallopian tubes, ovaries, and omentum are common locations of involvement for FATWOs (2, 3). FATWOs usually occur in the 50s; however, the age range for FATWOs is reported from 15 to 83 (3). FATWO is mainly considered a benign tumor; however, it might exhibit aggressive behavior in several cases. Less than 100 FATWO case reports have been published, among which a few reports indicated malignant presentations for FATWO (4). Tumor progression has been reported in approximately 11% of the cases 2 years after diagnosis. Nevertheless, optimal clinical management of recurrent or metastatic FATWO lacks comprehensive recommendations (2). In this article, we introduce an aggressive form of bilateral FATWO in association with recurrence and metastasis.

## Case Presentation

**Fig. 1:**
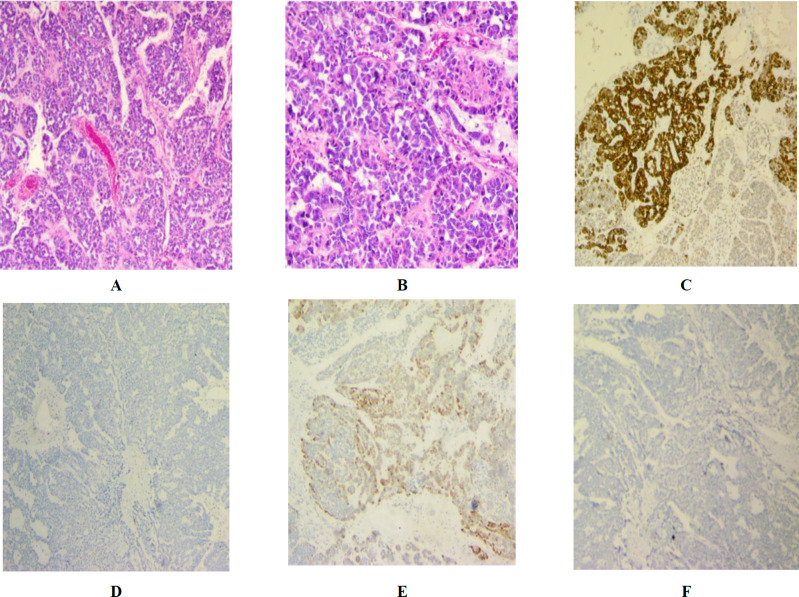
a) H&E section X40, b) H&E section X100, c) CK7, d) EMA, e) inhibin, f) PAX-8

A 35-year-old Iranian woman, G1P1L1, initially presented with an incidental ovarian mass in her abdominal ultrasound in 2019. Imaging studies revealed a left adnexal mass approximately 10 cm in diameter. Ovarian tumor markers including CA125, CEA, and CA19-9 were in normal limits. . Therefore, a left salpingo-oophorectomy and omental biopsy were performed. A morphologic study of the left adnexal mass revealed sheets of spindle cells associated with branching tubules and sieve-like patterns with cyst formation. Mitotic and nuclear pleomorphic figures were rare. The tumor’s invasion of the left fallopian tube serosa was evident. Omentum was also involved. Immunohistochemical studies were performed to exclude the mimics such as fallopian tube endometroid adenocarcinoma, granulosa cell tumor, and sertoli-leydig cell tumor. The neoplastic cells showed positive staining for calretinin, inhibin, WT1, CD56, vimentin, CK19, and CK7, while PAX-8, EMA, CD10, AFP and Glypican-3 staining were negative (Figure 1). 

Based on morphologic and immunohistochemical studies, an adnexal tumor with a probable Wolffian tube origin was diagnosed. The patient was followed up for 2 years till 2021, when imaging studies showed a right adnexal mass. A multiloculated cystic lesion with restriction and enhancement was detected in both right fallopian tube and ovary. Furthermore, magnetic resonance imaging (MRI) revealed a deep infiltrating endometriosis (DIE) with a 2.5 cm diameter in the cul de sac near the uterosacral ligament adjacent to the rectal fat plan. In laboratory evaluations, the CA-125 was 83, CA19-9 was 15, and CEA was 0.5.

In the second surgery, the right ovarian mass was removed and was submitted to the pathology lab for intraoperative consultation. The frozen sections suggested a Wolffian tumor. Therefore, right ovarian cystectomy, omental mass resection, and extraction of DIE were performed. A diagnosis of Wolffian tumor was confirmed in permanent sections. The excised tumor was 6cm in its greatest dimension with moderate nuclear atypia, 5/10hpf mitosis, ovarian surface involvement, and lymphovascular invasion. Furthermore, tumoral involvement was also observed in the omentum.

The Tumoral cells were positive for immunohistochemical markers, including CD10, CK7, ER, PR, and inhibin. Conversely, PAX-8, EMA, GATA-3, and TTF1 staining were negative. Based on the histopathological and clinical findings, a diagnosis of a bilateral adnexal tumor of Wolffian origin with malignant behavior was confirmed.

**Fig. 2:**
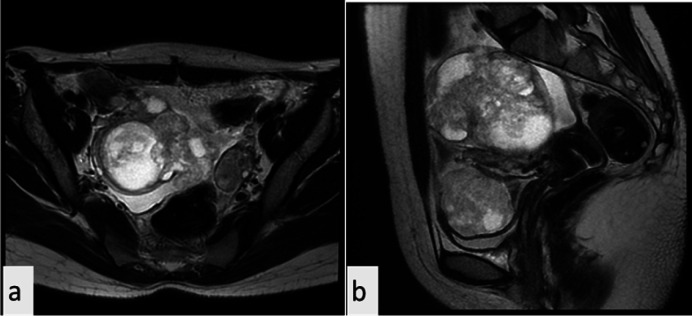
MRI study: a. Sagittal T2 images. b. AxialT2 images

The follow-up MRI showed adnexal bilateral multilocular cystic lesions with enhancement, which represented Ovarian-Adnexal Reporting & Data System (ORADS) 5 (Figure 2).

Consequently, a third surgical procedure was scheduled, during which the patient received comprehensive debulking surgery. This included total abdominal hysterectomy, right salpingo-oophorectomy, omentectomy, and lymphadenectomy.

Finally, a treatment plan was devised consisting of 6 rounds of adjuvant chemotherapy using paclitaxel (175 mg/m2) and carboplatinum (AUC5), followed by external pelvic radiotherapy by our multidisciplinary team. The patient is still under close observation, and no recurrences have been detected.

## Discussion

FATWO, which stands for Female Adnexal Tumor of Wolffian Origin, is a neoplasm with low potential for malignancy.; however, few cases of aggressive FATWOs have been reported to date (5). The majority of FATWOs exhibit a harmless demeanor and do not reoccur following surgical removal, nevertheless, malignant behavior might be seen in approximately one-fifth of Wolffian tumors. The median time for tumor recurrence was reported to be 48 months (7 to 96 months) (6, 7).

Our literature review showed that the metastasis and recurrence rate was 11% within 2 years of presentation (3, 8-10). The age of FATWO patients may range from 19 to 83 years, while the mean age of diagnosis is 50 years old (2, 6).

**Table 1:** Literature review

Author	Year of publish	Age	Clinical presentation	Primary tumor site	Tumor size	IHC	Recurrence site	Time to recurrence(months)	Patient’s status
“Taxy”	“1976”	“41”	“Abnormal uterine bleeding”	“Right broad ligament”	“8.5 cm”	-	“Liver”	“55”	“Alive”
“Abbot”	“1981”	“18”	“Acute abdomen”	“Right mesosalpinx”	“8.5 cm”	-	“Right adnexa, serosal surfaces of peritoneum”	“78”	“Dead 8y after”
“Hughesdon”	“1982”	“72”	“Constipation, urinary retention”	“Left ovary”	“14 cm”	-	“Pouch of Douglass”	“14”	“” Dead 14 m after”
“Brescia”	“1985”	“23”	“RLQ pain”	“Pararectal space”	“13 cm”	-	“The lower pole of the surgical incision, omentum” “Omentum, hepatic flexure” “Rt liver lobe”	“21” “36” “84”	“Alive”
“Prasad”	“1992”	“47”	“Tenesmus”	“Right ovary, broad ligament”	“12 cm”	“CK, EMA”	-	-	“Alive”
“Daya”	“1993”	“20”	“RLQ pain”	“Right lateral vaginal wall”	“12 cm”	-	“Site of previous surgery” “Paravaginal areas”	“24” “12”	
“Daya”	“1994”	“81”	“Abdominal distension, weight loss”	“Right broad ligament”	“20 cm”		-	-	“Dead 3m after”
“Sheyn”	“2000”	“60”	“Abdominal mass”	“Right mesosalpinx”	“11 cm”	“CAm 5.2, Vimentin”	“Liver”	“61”	“Not reported”
“Ramirez”	“2002”	“38”	“Enlarging Abdominal mass”	“Pelvis”	“17 cm”	“PR”	“Right anterior abdominal wall, liver, spleen, pelvis”	“4”	“Alive”
“Ramirez”	“2002”	“71”	“Incidental left adnexal mass on examination”	“Pelvis”	“16 cm”	« Calretinin, CK, Moc31, CK5/6, ER, PR »	Peritoneal implant, liver margin	“10”	“Alive”
“Atallah”	“2004”	“27”	“Incidental left adnexal mass on examination”	“Left broad ligament”	“11 cm”		“Peritoneal implants”	“27”	“Dead after 2 years”
“Steed”	“2004”	“15”	“Abdominal pain”	“Retroperitoneum, paravaginal broad ligament”	“14.2 cm”	“CK7, CK19, CAM 5.2, EMA, Vimentin”	“Broad ligament, uterosacral ligaments, abdominal wall”	“Less than 24”	“Alive”
“Halushkha”	“2004”	“34”	“Right-side pelvic pain”	“Right fallopian tube”	“5.8 cm”	“Pan CK, CAM 5.2, Calretinin, inhibin”	“Not reported”		“alive”
“Sivridis”	“2005”	“76”	“Abdominal pain, urinary retention”	“Right broad ligament”	“20 cm”	“Pan CK, Vimentin, S100, NSE”	“Not reported”		“Dead after 4 months”
“Tamiolakis”	“2007”	“75”	“Ascites, Urinary retention”	“Right broad ligament”	“4.7 cm”	“Pan CK, CAM 5.2, Calretinin, Inhibin”	“Left broad ligament”	“24”	“Not reported”
“Lesin”	“2009”	“60”	“Lower abdominal pain”	“Right broad ligament”	“8 cm”	“Not performed”	“Vaginal cuff”	“72”	“Alive”
“Syriac”	“2011”	“38”	“Right adnexal mass”	“Right broad ligament”	“12 cm”	“Pan CK, CK7, WT1, Calretinin”	“Left ovary”	“36”	“Not reported”
“Heller”	“2011”	“24”	“Pelvic pain”	“Left broad ligament”	“4 cm”	“Calretinin, vimentin, CK7, Vimentin”	“Appendix, small bowel serosa, omentum, posterior bladder peritoneum, broad ligament”	“1.5 “	“Lost to follow up”
“Liu”	“2014”	“24”	“Pelvic pain”	“Left broad ligament”	“Not reported”	“ER, Calretinin, CK, vimentin, Inhibin”	“Serosa of the appendix”	“1”	“Not reported”
“Deshimaru”	“2014”	“30”	“Right ovarian mass on examination and ultrasound”	“The right fallopian tube, broad ligament”	“5 cm”	« Calretinin, Inhibin, CD10, Vimentin, Desmin, CD34 »	“Tumor implants on bowel serosal surface, omentum, left ovary, pouch of Douglas”	“4”	“Dead 3y after”
“Deen”	“2007”	“81”	“Post-menopausal bleeding, pelvic mass in imaging”	“Right ovary”	“18 cm”	“Vimentin, Calretinin, alpha-inhibin, Chromogranin A, CD56, MIB1”	“Right adnexa, paravaginal area”	“7”	“Not reported”
“Kwon”	“2016	“52”	“Pelvic pain”	“Left ovary hilum”	“8 cm”	“D2-40, Calretinin, CK, CD10, CD56, Vimentin, CK7, Mucicarmine”	« Right side cul de sac »	« 9 «	« Lost to follow up »
“Qiu”	“2017”	“53”	“Abdominal distension”	“Left mesosalpinx”	“10 cm”	“Inhibin A, Calretinin, ER, PR, CD99, PAX-2, CK “	“Abdomen and pelvic cavity”	“24”	“Dead 83d after second surgery”
“Wakayama”	“2017”	“37”	“Lower abdominal pain”	“Left fallopian tube”	“7 cm”	“CK7, Vimentin, Inhibin, Calretinin”	“Peritoneal implants”	“17”	“alive”
“Hong”	“2018”	“50”	“Lower abdominal pain”	“Left ovary”	“17 cm”	« ER,PR,CK7, EMA, CD10 »	« Not reported »		« Alive »
“Sinha(11)”	“2021”	“28”	“LLQ pain”	“Left mesosalpinx”	“3 cm”	“Pan CK, CK7, CD10, Calretinin, Inhibin, ER, PR, C-kit”	“Seeding and recurrence in the umbilical port side “	“8”	“Alive”
“Chen(12)”	“2021”	“75”	“Right adnexal mass”	“Broad ligament”	“8 cm”	« CK7, Vimentin, EMA, TTF1, CD10, PAX-8, PAX-2, P16 »	“The right pelvic cavity adhered to the right broad ligament”	“36”	“Alive”
Present case	2023	35	Left adnexal mass; incidental finding in ultrasound	Left broad ligament	10 cm	Calretinin, inhibin, WT1, CD56, Vimentin, CK19, CK7	Right fallopian tube and ovary	24	Alive

FATWOs arise from the regressing Wolffian system. Tumor pathogenesis includes STK11, APC, and MDB4 mutations as well as KMT2D variants, which are of unknown biological significance (6). 

FATWOs usually manifest on one side and commonly develop as masses within the broad ligament or as hanging growths with a thin stalk from adnexal structures. Currently, there are no identified immunohistochemical markers specific for FATWOs.

The main differential diagnosis for FATWO is endometrioid carcinoma with a FATWO-like pattern. However, unlike FATWO, endometrioid carcinomas present with squamous morules, glandular cells with luminal polarization, and intraluminal mucin that predominantly involve the fallopian tube and show positive immunoreaction with PAX-8, EMA, ER, and PR (7). 

When distinguished from FATWO, mesonephric carcinoma and mesonephric-like carcinomas are considered due to the presence of small tubules containing luminal eosinophilic material. Unlike FATWO, which lacks a specific anatomical location or a sieve-like microscopic pattern, mesonephric carcinomas typically exhibit strong positivity for GATA-3 in immunohistochemical testing. Conversely, FATWO displays an opposite phenotype (7). 

Based on reports from the literature, no single histologic feature indicates malignancy, and, in some cases, recurrences occurred even long after the diagnosis. Unlike benign forms, malignant FATWOs mainly present with extrauterine spread, recurrence, or distant metastasis. Our case represented a malignant FATWO as evidenced by metastasis to the right ovary since bilateral FATWO is extremely rare. The tumor in the first pathology specimen invaded the fallopian tube wall and its lumen. There was evidence of lymphovascular invasion and omentum involvement, which are indicative of malignant behavior of the tumor.

The most common sites of metastasis include the liver and lung (13). Recurrence occurs in most cases undergoing single tumor resection (14). Clinical manifestations have not been associated with histomorphological features of FATWO (15). The known features that indicate malignant potential in FATWOs include necrosis, capsular invasion, high mitotic rate, nuclear pleomorphism, CD117 positive staining, and overexpression of Ki-67 (16). 

Since FATWO is uncommon, there is currently no established best way to manage it. However, the most successful treatment for primary FATWO is complete tumor removal, which includes hysterectomy, bilateral salpingo-oophorectomy, and debulking surgery. It is worth noting that most tumor recurrences occur in patients who initially underwent conservative treatments like cystectomy or simple tumor removal, as was the case in our situation. (3, 15, 16). Despite some cases of remission or partial remission after certain adjuvant treatments, the precise impact of radiotherapy, chemotherapy, hormone therapy, and molecular-targeting therapy on malignant FATWO remains unknown. Hence, it is essential to develop an appropriate treatment plan for individuals diagnosed with malignant FATWO.

Various chemotherapy protocols have been employed in management of recurrent and metastatic FATWO. The most frequent chemotherapy regimen is a combination of paclitaxel and carboplatin. Tyrosine kinase inhibitors, including imatinib, were considered in the patients with c-Kit+ tumors (2, 3). Nevertheless, this medication proved to be ineffective in achieving the desired outcome. Additional research is required to ascertain the efficacy of various chemotherapy protocols in treating malignant FATWO.

## Conclusion

The FATWO, a rare gynecologic tumor believed to stem from the Wolffian duct, is an uncommon condition, with its malignant variant being exceptionally rare. Diagnosis of FATWO requires a thorough examination of the immunohistochemical panel and histomorphologic studies. No histologic criteria or serum biomarkers have yet been determined to predict the prognosis of FATW. Therefore, periodic routine follow-up is essential for all FATWO patients. 

Therapeutic options have emphasized complete debulking surgery. There is controversy in performing chemotherapy and radiation therapy as an adjuvant treatment in recurrent and malignant FATWOs. A systematic review of all published case reports and patient follow-up will help determine the most effective treatment approach. 

## Abbreviations

FATWOs: Wolffian adnexal tumors, CA: Cancer Antigen, MRI: magnetic resonance imaging, DIE: Deep infiltrating endometriosis,

## References

[1] Kariminejad MH, Scully RE (1973). Female adnexal tumor of probable Wolffian origin A distinctive pathologic entity. Cancer.

[2] Chen Q, Shen Y, Xie C (2021). Recurrent and metastatic female adnexal tumor of probable Wolffian origin: A case report and review of the literature. Medicine.

[3] Bunnell ME, Donovan BM, Parrack PH, Muto MG, Horowitz NS, Leung SOA (2020). Female adnexal tumor of probable Wolffian Origin-A report of two cases at one institution. Gynecol Oncol Rep.

[4] Liu L, Fang Q, Xing Y (2018). Female adnexal tumor of probable Wolffian origin arising from mesosalpinx: a case report and review. J Obstet Gynaecol Res.

[5] Syriac S, Durie N, Kesterson J, Lele S, Mhawech-Fauceglia P (2011). Female Adnexal Tumor of Probable Wolffian Origin (FATWO) With Recurrence 3 Years Postsurgery: C-kit Gene Analysis and a Possibility of a New Molecular Targeted Therapy. Int J Gynecol Pathol.

[6] Herrington CS (2020). WHO Classification of Tumours Female Genital Tumours: International Agency for Research on Cance.

[7] Nucci MR, Parra-Herran C (2019). Gynecologic Pathology E-Book: A Volume in the Series: Foundations in Diagnostic Pathology.

[8] Harada O, Ota H, Takagi K, Matsuura H, Hidaka E, Nakayama J (2006). Female adnexal tumor of probable Wolffian origin: Morphological, immunohistochemical, and ultrastructural study with c‐kit gene analysis. Pathol Int.

[9] Hong S, Cui J, Li L, Buscema J, Liggins C, Zheng W (2018). Malignant female adnexal tumor of probable Wolffian origin: case report and literature review. Int J Gynecol Pathol.

[10] Atallah D, Rouzier R, Voutsadakis I, Sader-Ghorra C, Azoury J, Camatte S (2004). Malignant female adnexal tumor of probable Wolffian origin relapsing after pregnancy. Gynecol Oncol.

[11] Sinha R, Bustamante B, Tahmasebi F, Goldberg GL (2021 ). Malignant Female Adnexal Tumor of Probable Wolffian Origin (FATWO): A case report and review for the literature. Gynecol Oncol Rep.

[12] Chen Q, Shen Y, Xie C (2021). Recurrent and metastatic female adnexal tumor of probable Wolffian origin: A case report and review of the literature. Medicine (Baltimore).

[13] Ramirez PT, Wolf JK, Malpica A, Deavers MT, Liu J, Broaddus R (2002). Wolffian duct tumors: case reports and review of the literature. Gynecologic oncology.

[14] Kommoss F, Oliva E, Bhan AK, Young RH, Scully RE (1998). Inhibin expression in ovarian tumors and tumor-like lesions: an immunohistochemical study. Mod Pathol.

[15] Wakayama A, Matsumoto H, Aoyama H, Saio M, Kumagai A, Ooyama T (2017). Recurrent female adnexal tumor of probable Wolffian origin treated with debulking surgery, imatinib and paclitaxel/carboplatin combination chemotherapy: a case report. Oncol Lett.

[16] Lešin J, Forko-Ilić J, Plavec A, Planinić P (2009). Management of Wolffian duct tumor recurrence without chemotherapy. Arch Gynecol Obstet.

